# Association of beta‐hydroxybutyrate with development of heart failure: Sex differences in a Dutch population cohort

**DOI:** 10.1111/eci.13468

**Published:** 2020-12-18

**Authors:** Jose L. Flores‐Guerrero, Berend Daan Westenbrink, Margery A. Connelly, James D. Otvos, Dion Groothof, Irina Shalaurova, Erwin Garcia, Gerjan Navis, Rudolf A. de Boer, Stephan J. L. Bakker, Robin P. F. Dullaart

**Affiliations:** ^1^ Department of Internal Medicine Division of Nephrology University of Groningen University Medical Center Groningen Groningen The Netherlands; ^2^ Department of Cardiology University of Groningen University Medical Center Groningen Groningen The Netherlands; ^3^ Laboratory Corporation of America Holdings (LabCorp) Morrisville NC USA; ^4^ Department of Internal Medicine Division of Endocrinology University of Groningen University Medical Center Groningen Groningen The Netherlands

**Keywords:** beta‐hydroxybutyrate, heart failure, ketone bodies, sex differences

## Abstract

**Background:**

In the failing heart, energy metabolism is shifted towards increased ketone body oxidation. Nevertheless, the association of beta‐hydroxybutyrate (β‐OHB) with development of heart failure (HF) remains unclear. We investigated the association between plasma β‐OHB and the risk of HF in a prospective population‐based cohort.

**Design:**

Plasma β‐OHB concentrations were measured in 6134 participants of the PREVEND study. Risk of incident HF with reduced (HFrEF) or preserved (HFpEF) ejection fraction was estimated using multivariable‐adjusted Cox regression models.

**Results:**

During median follow‐up for 8.2 years, 227 subjects were diagnosed with HF (137 with HFrEF; 90 with HFpEF). Cox regression analyses revealed a significant association of higher β‐OHB concentrations with incident HF (HR per 1 standard deviation increase, 1.40 (95% CI: 1.21‐1.63; *P* < .001), which was largely attributable to HFrEF. In women, the hazard ratio (HR) for HFrEF per 1 standard deviation increase in β‐OHB was 1.73 (95% confidence interval (CI): 1.17‐2.56, *P* = .005) in age, BMI, type 2 diabetes, hypertension, myocardial infarction, smoking, alcohol consumption, total cholesterol, HDL‐C, triglycerides, glucose, eGFR and UAE adjusted analysis. In men, in the same fully adjusted analysis, the HR was 1.14 (CI: 0.86‐1.53, *P* = .36) (*P* < .01 for sex interaction). In N‐terminal pro‐brain natriuretic peptide (NT‐proBNP)‐stratified analysis, the age‐adjusted association with HF was significant in women with higher NT‐proBNP levels (*P* = .008).

**Conclusions:**

This prospective study suggests that high plasma concentrations of β‐OHB are associated with an increased risk of HFrEF, particularly in women. The mechanisms responsible for the sex differences of this association warrant further study.

## 1 INTRODUCTION

Heart failure (HF) is a global pandemic. In the US, the prevalence of HF is expected to increase by 46% from 2012 to 2030.^1^ Hence, it is clinically relevant to better understand the metabolic abnormalities underlying this complex clinical syndrome. In addition, considering that 55% of the total cardiovascular mortality in women can be attributed to HF,^2^ elucidation of sex‐specific risk factors for HF may lead new targets for its prevention.

Due to its high energy consumption and limited ability to store ATP, the heart is highly dependent on a continuous supply and efficient oxidation of exogenous metabolites. In patients with HF, metabolic roadblocks in fat and carbohydrate metabolism reduce the myocardial capacity to generate ATP, resulting in a myocardial energy deficit.^3^

Ketone bodies are endogenous metabolites that are mainly produced by the liver, in particular under conditions of prolonged fasting, insulin deprivation and extreme exercise.^3^ Circulating ketone bodies concentrations and the cardiac uptake of ketone bodies are increased in patients with HF, both in those with reduced (HFrEF) and with preserved (HFpEF) ejection fraction.^4^, ^5^ Experimental evidence also indicates that cardiac energy metabolism in the failing heart re‐programmes towards increased oxidation of ketone bodies.^6^ Furthermore, sodium‐glucose co‐transporter 2 inhibitors (SGLT2i) are known to increase circulating ketone levels and reduce the rates of hospitalization for HF both in patients with diabetes^7^, ^8^ and without diabetes.^9^ It has been proposed that increased ketone oxidation in the heart is responsible for this effect.^6^ Taken together, higher concentrations of ketone levels in the circulation of heart failure patients appear to coincide with an adaptive increase in myocardial ketone oxidation.

Beta‐hydroxybutyrate (β‐OHB) is the most abundant ketone body in the circulation and has become subject of study in pathological conditions like cardiovascular disease (CVD),^3^, ^10^ HF^11^ and chronic kidney disease (CKD).^12^ Early findings have pointed to increased plasma concentrations of β‐OHB in patients with chronic HF.^13^ In addition, it has been reported that HF patients present higher concentrations of acetone in the exhaled breath, proportional to HF severity.^14^

Considering the possible implications of ketone body metabolism in the failing heart, the present study aimed to explore the longitudinal association of β‐OHB with incidence of preserved and reduced ejection fraction HF in participants enrolled in the Prevention of Renal and Vascular End‐stage Disease (PREVEND), a prospective Dutch population‐based cohort study.

## 2 METHODS

### 2.1 Study cohort

The PREVEND Study is a prospective population‐based cohort study conducted in Groningen, the Netherlands. The design of the PREVEND Study has been described in detail elsewhere.^15^ Briefly, from 1997 to 1998, all residents from Groningen, excluding pregnant women and people with type 1 diabetes or T2D using insulin, aged 28‐75 years were invited to participate; a total of 8592 individuals completed an extensive examination.

For the present analysis, data from participants who completed the second screening round (2001‐2003; n = 6894) were used, excluding those with missing values for β‐OHB concentrations (n = 359), as well as those who met any of the following conditions: known HF diagnosis at baseline, were not fasting at the time blood samples were taken, had insulin concentrations higher than > 25 mIU/L, because they presumably were nonfasting (n = 401). This left a cohort of 6134 participants with complete information for the current analysis (Figure S1). The protocol for the PREVEND study was approved by the local ethics committee. Reporting of the study conforms to broad EQUATOR guidelines.^16^ The investigation conforms with the principles outlined in the Declaration of Helsinki, and all participants provided written informed consent to participate in the study.

### 2.2 Ascertainment of heart failure

Participants were followed from the date of the baseline visit until end of follow‐up (census date: January first, 2011).

Participants with a new diagnosis of HF were identified using criteria described in the HF Guidelines of the European Society of Cardiology as published in 2012, and the methodology for HF diagnosis employed in the present study has been published in detail.^17^ Briefly, HF was diagnosed if patients who presented with typical symptoms (ie breathlessness, ankle swelling, fatigue, orthopnea and paroxysmal nocturnal dyspnoea), and/or signs (ie elevated jugular venous pressure, pulmonary crackles and displaced apex beat), combined with objective measures of cardiac dysfunction.

A committee of seven independent experts in the field of HF evaluated all cases suspected for the diagnosis of new‐onset HF. All cases of new‐onset HF were validated by two different experts in the field of HF by reviewing anonymized clinical charts, hospitalization and physician office records in order to ascertain the incidence of heart failure. In case of difference of opinion about an individual case, the committee made a joint decision.^17^ Based on left ventricular ejection fraction (LVEF) at the time of diagnosis, HF was classified as HFrEF or HFpEF (LVEF ≤ 40 or ≥ 50%, respectively). ^18^ LVEF, data were available through imaging techniques, such as echocardiography, MRI or radionuclide ventriculography.

### 2.3 Laboratory measurements

Venous blood samples were taken from participants after an overnight fast and 15 minutes of rest prior to sample collection at the second screening round. All blood samples were taken between 8:00 and 10:00 am. Ethylenediaminetetraacetic acid (EDTA)‐anticoagulated plasma samples were prepared by centrifugation at 4°C and were stored at −80°C until analysis.

Plasma β‐OHB was measured using a Vantera Clinical Analyzer (LabCorp), a fully automated, high‐throughput, 400 MHz proton (1H) nuclear magnetic resonance (NMR) spectroscopy platform. Plasma samples were prepared on board the instrument and automatically delivered to the flow probe in the NMR spectrometer's magnetic field. Data acquisition on the Vantera and spectra data processing have been reported in greater detail elsewhere.^19^ Leucine was measured by NMR spectroscopy as reported.^20^

Total cholesterol, triglycerides, insulin, serum creatinine and serum cystatin C were measured using standard protocols.^21^, ^22^ Urinary albumin excretion (UAE) was measured as described in two 24‐hour urine collections, and the results were averaged for analysis.^23^ Fasting plasma glucose was measured by dry chemistry (Eastman Kodak). N‐terminal pro‐B‐type natriuretic peptide (NT‐proBNP) was measured in plasma on an Elecsys™ 2010 analyser. The estimated glomerular filtration rate (eGFR) was calculated using the Chronic Kidney Disease Epidemiology Collaboration CKD‐EPI combined creatinine‐cystatin C equation.

### 2.4 Clinical measurements

During two outpatient visits, baseline data were collected on demographics, lifestyle factors, anthropometric measurements and medical history. Systolic and diastolic blood pressure values were recorded as the means of the last two recordings of the second visit, using an automatic Dinamap XL Model 9300 series device.

### 2.5 Statistical analysis

Data are presented as the means (standard deviation, SD) or medians (interquartile range, IQR) for continuous variables and percentages for categorical variables. Cross‐sectional associations at baseline were assessed by one‐way analysis of variance for normally distributed data, Kruskal‐Wallis test for skewed distributed data and by *χ* ^2^ test for categorical variables. The analyses were conducted using sex‐stratified tertiles of plasma β‐OHB in order to have sex‐balanced tertiles and decrease the heterogeneity of risk within groups due to a sex effect. Baseline concentrations of plasma β‐OHB in men and women were compared using the Wilcoxon rank‐sum test.

For the prospective analysis, time‐to‐event Cox proportional hazards models were used to assess the hazard ratio (HR) and 95% CI of HF incidence among the 5963 participants with full information at baseline. HRs were calculated in models adjusted for age, sex, BMI > 30 kg/m^2^, T2D, hypertension, myocardial infarction, tobacco and alcohol consumption, total cholesterol, high‐density lipoprotein cholesterol (HDL‐C), triglycerides, glucose, leucine, N‐terminal pro‐brain natriuretic peptide (NT‐proBNP), eGFR and UAE. The proportionality of hazards assumption was tested through the evaluation of independence between scaled Schoenfeld residuals with time for each variable and for every model as a whole. Additionally, interactions for sex, age, menopause stage, hormone replacement therapy, alcohol consumption and smoking status were tested, and sensitivity analysis excluding participants with T2D were conducted. Two‐sided *P* values < .05 were considered to be significant.

All statistical analyses were performed with R language for statistical computing software (v. 3.6.2; R Foundation for Statistical Computing).

## 3 RESULTS

### 3.1 Baseline characteristics

Characteristics of the 6134 participants at baseline are shown in Table 1. Among the participants, 3028 (49.3%) were men and the mean age of the population was 53.3 ± 12.0 years. The median β‐OHB concentration was 121.4 (IQR, 92.8‐169.1) µmol/L (Table 1) (women: 123.2 (IQR, 93.5‐174.9) µmol/L; men: 119.2 (IQR, 92.1‐ 163.8) µmol/L; *P* < .01). Subjects in the highest tertile of β‐OHB concentration were more likely to be older, have higher BMI, systolic and diastolic blood pressure, and present with a higher prevalence of T2D. Additionally, these subjects had higher concentrations of total cholesterol, glucose, leucine and elevated UAE. The percentages of a family history of T2D were similar among the different tertiles of β‐OHB (Table 1). Concentrations of β‐OHB at baseline were significantly different between individuals who develop HF and those who did not develop HF during the follow‐up. (Table S1).

**TABLE 1:** Participant characteristics by sex‐stratified tertiles of beta‐hydroxybutyrate (β‐OHB) in PREVEND participants (n = 6134)

	All Participants	Tertiles of β‐OHB, μmol/L			*P*‐value*
		T1	T2	T3	
		♀<102.3	♀102.3‐152.3	♀>152.3	
		♂ <100.9	♂ 100.9‐145.3	♂ >145.3	
Participants, n	6134	2057	2021	2056	
Sex, men, %	49.3	49.3	49.3	49.3	.99
Age, y	53.3 ± 12.0	50.3 ± 11.1	54.4 ± 11.8	55.9 ± 12.5	<.001
BMI, kg/m^2^	26.4 ± 4.1	25.8 ± 3.7	26.9 ± 4.2	26.7 ± 4.3	<.001
SBP, mm Hg	125.8 ± 18.6	122.2 ± 16.6	126.6 ± 18.4	128.7 ± 20.1	<.001
DBP, mm Hg	73.2 ± 9.0	70.1 ± 8.7	73.5 ± 8.7	74.1 ± 9.5	<.001
Heart rate, bpm	68.3 ± 10.0	66.9 ± 9.5	68.2 ± 9.4	69.8 ± 10.8	<.001
T2D, yes, %	5.3	1.8	5.2	9.0	<.001
HT, yes, %	30.6	22.5	33.0	36.5	<.001
Myocardial infarction, yes, %	1.4	1.1	1.4	1.6	.41
Atrial fibrillation history, yes, %	1.7	0.9	1.6	2.6	<.001
Cancer history, yes, %	4.3	4.6	4.4	3.8	.35
Smoking status					.04
Never, %	28.5	30.6	27.4	27.9	
Former, %	42.2	39.5	44.0	42.2	
Current, %	27.7	28.1	27.6	27.6	
Alcohol consumption					<.001
<1 drinks/week, %	24.4	24.7	24.4	24.1	
1‐7 drinks/week, %	48.2	49.9	50.3	44.3	
>7 drinks/week, %	26.4	24.2	24.5	30.3	
ACE inhibitor drugs,%	8.3	5.8	8.6	10.5	<.001
ARBs drugs, %	2.0	1.6	2.0	2.6	.06
Beta blocker drugs, %	9.3	6.7	11.0	10.3	<.001
Lipid‐lowering drugs, %	7.8	5.9	8.8	8.6	.003
β‐OHB, μmol/L	121.4(92.8‐169.1)	82.8(71.0‐92.9)	121.2(111.7‐122.7)	206.3(169.0‐282.1)	<.001
Glucose, mmol/L	5.0 ± 1.2	4.8 ± 0.7	5.0 ± 1.0	5.2 ± 1.4	<.001
Insulin, mU/L	8.0(5.7‐11.7)	7.2(5.2‐10.0)	8.7(6.2‐12.6)	8.2(5.8‐12.3)	<.001
TG, mmol/L	1.1(0.8‐1.5)	1.0(0.7‐1.4)	1.2(0.9‐1.7)	1.1(0.8‐1.7)	<.001
TC, mmol/L	5.4 ± 1.0	5.3 ± 1.0	5.5 ± 1.0	5.4 ± 1.0	<.001
HDL‐C, mmol/L	1.2 ± 0.3	1.3 ± 0.3	1.2 ± 0.3	1.2 ± 0.3	<.001
Leucine, μmol/L	127.7 ± 28.1	124.2 ± 24.7	129.7 ± 27.1	129.3 ± 31.6	<.001
NT‐proBNP, ng/L	44.0(24.0‐84.0)	39.0(21.0‐70.0)	42.0(23.0‐81.0)	52.5(28‐105.0)	<.001
eGFR, mL/min/1.73 m^2^	92.2 ± 17.1	96.0 ± 15.8	91.5 ± 17.0	89.3 ± 17.7	<.001
UAE, mg/24 h	8.6(6.0‐15.5)	8.0(5.9‐12.9)	8.8(6.0‐16.2)	9.3(6.2‐19.1)	<.001

Note

Continuous variables are reported as mean ± SD, median (interquartile range) and categorical variables are reported as percentage.

Abbreviations: ACE, Angiotensin converting enzyme; ARBs, Angiotensin II receptor blockers; BMI, body mass index; bpm, beats per minute, T2D, type 2 diabetes; DBP, diastolic blood pressure; GFR, estimated glomerular filtration rate; HDL‐C, high‐density lipoprotein cholesterol; HT, hypertension; NT‐proBNP, N‐terminal pro‐brain natriuretic peptide; SBP, systolic blood pressure; TC, total cholesterol; TG, triglycerides; UAE, urinary albumin excretion; β‐OHB, beta‐hydroxybutyrate.

* P values represent the significance of difference across the tertiles of plasmatic β‐OHB. P values were determined using a one‐way analysis of variance for normally distributed data, Kruskal‐Wallis test for skewed distributed data, and chi‐square test for categorical data.

### 3.2 Associations at baseline

The associations of β‐OHB concentrations with other variables of interest were evaluated with multivariable linear regression analyses. In a multivariable model, including all the variables presented in Table 1, β‐OHB remained positively associated with the following variables: age, systolic blood pressure, T2D, alcohol consumption, total cholesterol, leucine and NT‐proBNP. β‐OHB was negatively associated with male sex and total cholesterol (*P* < .05). (Table S2).

### 3.3 Longitudinal analyses on overall HF

During a median follow‐up period of 8.5 years (IQR, 8.0‐9.0), 227 participants developed HF (149 men and 78 women). Among them, 137 participants developed HFrEF (100 men and 37 women) and 90 participants developed HFpEF (49 men and 41 women).

**FIGURE 1:**
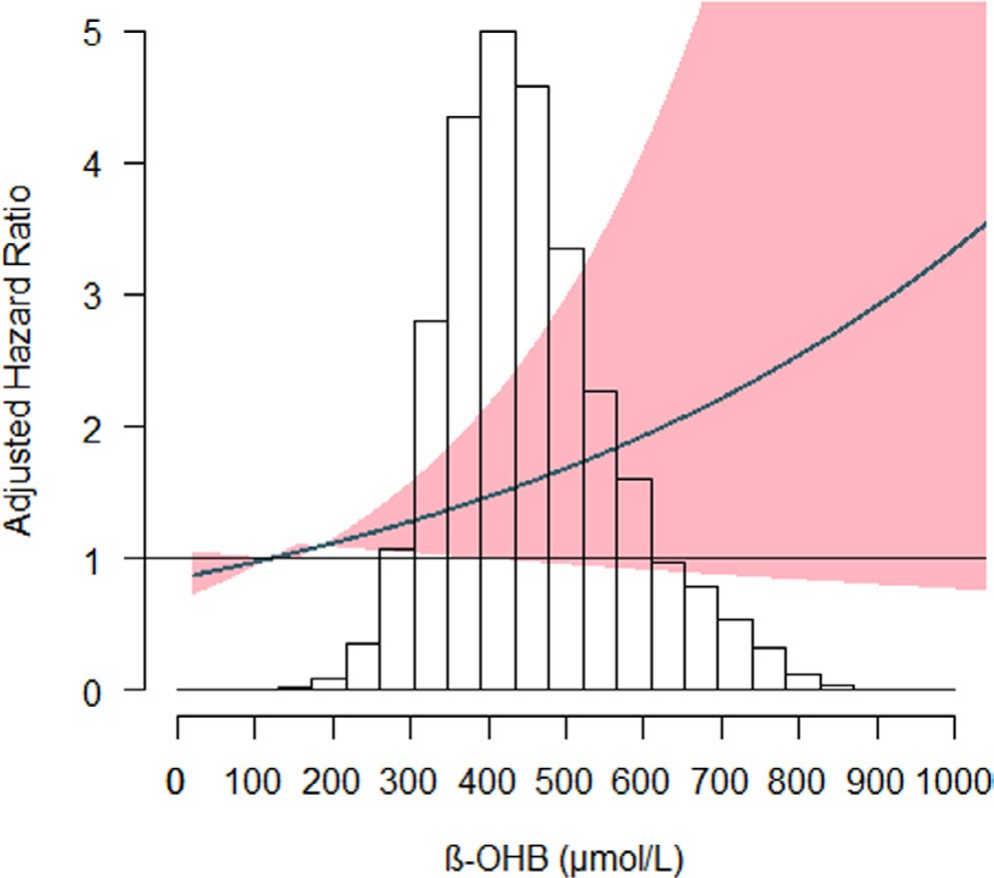
Association between β‐OHB plasma concentrations and HF in women. The *x*‐axis depicts the plasma concentration of β‐OHB in women of the PREVEND cohort, and the *y*‐axis depicts the new‐onset heart failure hazard ratio after adjustment for: age, BMI > 30 kg/m^2^, T2D, hypertension, myocardial infarction, smoking, alcohol consumption, total cholesterol, HDL‐C, triglycerides, glucose, eGFR and UAE. The shaded areas indicate the upper and lower 95% CI. HF, Heart Failure; β‐OHB, beta‐hydroxybutyrate

**FIGURE 2:**
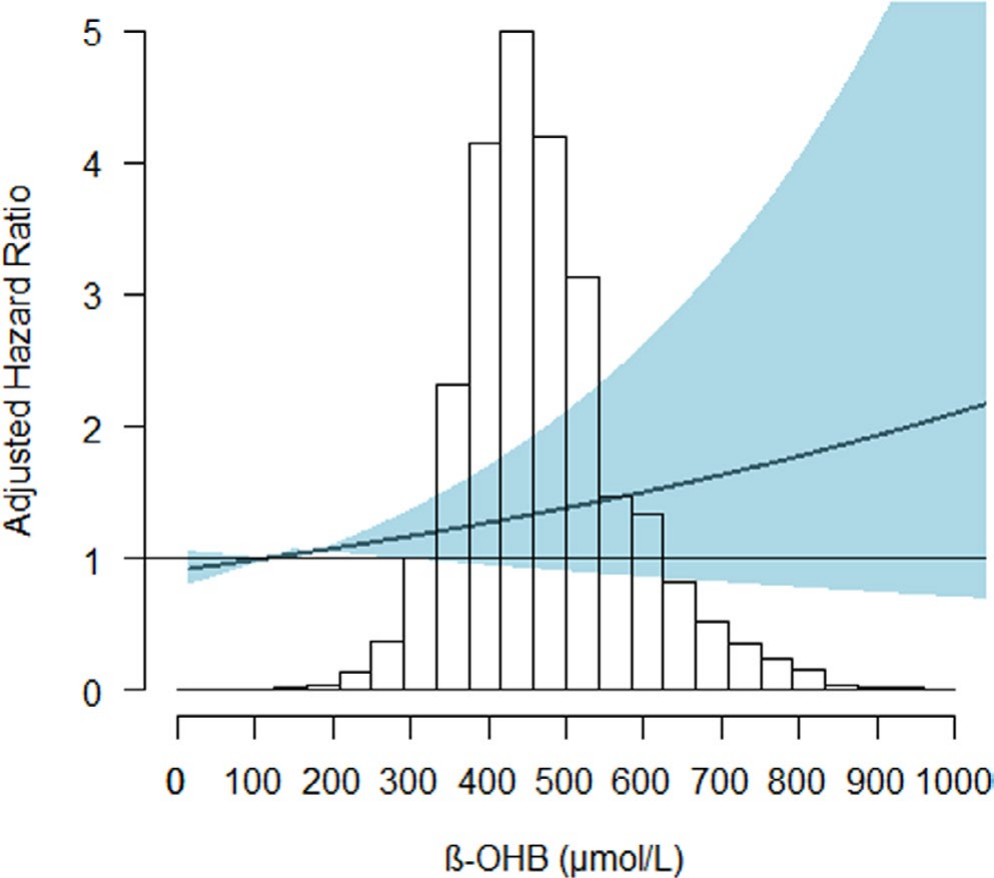
Association between β‐OHB plasma concentrations and HF in men. The *x*‐axis depicts the plasma concentration of β‐OHB in men of the PREVEND cohort, and the *y*‐axis depicts the new‐onset heart failure hazard ratio after adjustment for: age, BMI > 30 kg/m^2^, T2D, hypertension, myocardial infarction, smoking, alcohol consumption, total cholesterol, HDL‐C, triglycerides, glucose, eGFR and UAE. The shaded areas indicate the upper and lower 95% CI. HF, Heart Failure; β‐OHB, beta‐hydroxybutyrate

Cox regression analyses revealed that, 1 SD increase in β‐OHB concentration was associated with a 40% increase in the risk of total HF in the crude model (HR per 1 SD increase, 1.40 (95% CI: 1.21‐1.63; *P* < .001) (Table 2). This association did not remain statistically significant in the fully adjusted model (HR per one SD increase: 1.03, 95% CI: 0.83‐1.28; *P* = .79) (Table 2). There was a significant sex interaction on the association of β‐OHB with risk of HF (*P* < .01). Sex‐specific differences are graphically illustrated in Figure 1 and Figure 2.

**TABLE 2:** Association of β‐OHB with HF by sex‐stratified tertiles of plasma concentrations of β‐OHB

Events/Participants	T1	T2		T3		β‐OHB Per 1 SD Increment	
♀	14/1042	25/1023		39/1041		78/3106	
♂	30/1015	45/998		74/1015		149/3028	
		HR (95% CI)	*P*‐value	HR (95% CI)	*P*‐value	HR (95% CI)	*P*‐value
Crude model							
All	(ref)	1.64 [1.12‐2.40]	.01	2.36 [1.66‐3.36]	<.001	1.40 [1.21‐1.63]	<0.001
♀	(ref)	1.54 [0.79‐2.98]	.20	2.38 [1.29‐4.42]	.005	1.40 [1.12‐1.75]	0.003
♂	(ref)	1.66 [1.04‐2.65]	.03	2.40 [1.56‐3.69]	<.001	1.48 [1.21‐1.81]	<0.001
Model 1							
All	(ref)	1.26 [0.86‐1.85]	.23	1.59 [1.11‐2.27]	.01	1.21 [1.03‐1.42]	0.01
♀	(ref)	1.28 [0.66‐2.50]	.46	1.87 [1.00‐3.49]	.04	1.30 [1.02‐1.64]	0.03
♂	(ref)	1.21 [0.75‐1.94]	.42	1.43 [0.92‐2.21]	.11	1.19 [0.96‐1.48]	0.12
Model 2							
All	(ref)	1.18 [0.80;1.74]	.43	1.49 [1.04;2.15]	.03	1.22 [1.03;1.44]	0.02
♀	(ref)	1.33 [0.68;2.61]	.40	2.12 [1.12;4.01]	.02	1.40 [1.08;1.82]	0.01
♂	(ref)	1.03 [0.63;1.67]	.91	1.18 [0.75;1.86]	.46	1.15 [0.91;1.44]	0.23
Model 3							
All	(ref)	1.22 [0.81;1.83]	.34	1.42 [0.96;2.09]	.08	1.18 [0.99;1.41]	0.05
♀	(ref)	1.37 [0.66;2.85]	.40	2.12 [1.06;4.25]	.03	1.53 [1.11;2.13]	0.01
♂	(ref)	1.08 [0.65;1.79]	.78	1.17 [0.72;1.89]	.52	1.09 [0.89;1.34]	0.39
Model 4							
All	(ref)	0.70 [0.42;1.16]	.16	0.90 [0.57;1.43]	.65	1.03 [0.83;1.28]	0.79
♀	(ref)	0.98 [0.38;2.54]	.96	1.42 [0.57;3.53]	.45	1.36 [0.95;1.96]	0.99
♂	(ref)	0.62 [0.33;1.17]	.14	0.80 [0.45;1.40]	.42	1.06 [0.86;1.32]	0.56

Note

Model 1. Age + sex (sex‐specific analyses are adjusted for age only).

Model 2. Age + sex + BMI + T2D + hypertension + myocardial infarction + smoking + alcohol consumption.

Model 3. Age + sex + BMI + T2D + hypertension + myocardial infarction + smoking + alcohol consumption + total cholesterol + HDL‐C + triglycerides + leucine + glucose + eGFR + UAE.

Model 4. Age + sex + BMI + T2D + hypertension + myocardial infarction + smoking + alcohol consumption + total cholesterol + HDL‐C + triglycerides + leucine + glucose + eGFR + UAE + history of atrial fibrillation + heart rate + NT‐proBNP.

Abbreviations: BMI, body mass index; eGFR, estimated glomerular filtration rate; HDL‐C, high‐density lipoprotein cholesterol; HF, heart failure; NT‐proBNP, N‐terminal pro‐brain natriuretic peptide; T2D, type 2 diabetes; UAE, urinary albumin excretion.

The Cox regression analysis restricted to women showed that higher β‐OHB concentrations (top tertile) were associated with an increased risk of HF after adjustment for total cholesterol, HDL‐C, triglycerides, leucine, glucose, eGFR and UAE (model 3, Table 2) (HR: 1.47, 95% CI: 1.11‐1.94, *P* = .006). This association did not change in women after further adjustment for menopause stage and hormone replacement therapy (HR: 1.39, 95% CI: 1.02;1.91, *P* = .03). Nevertheless, the association did not remain after further adjustment for atrial fibrillation, heart rate and NT‐proBNP (model 4, Table 2). In men, the association was significant in the crude model, but not after adjustment for age and sex or other covariables (HR: 1.19, 95% CI: 0.96‐1.48, *P* = .12) (Table 2).

### 3.4 Longitudinal analyses on HFrEF

There was an association between β‐OHB plasma concentration and risk of HFrEF in both sexes, but after adjustment for age , BMI, T2D, hypertension, myocardial infarction, smoking, alcohol consumption, lipids, leucine, glucose, eGFR and UAE, the association remained significant only in women (HR per 1 SD increase: 1.83, 95% CI: 1.16‐2.91; *P* = .01), though not in men (HR per 1 SD increase: 1.13, 95% CI: 0.88‐1.46; *P* = .09) (Table 3).

**TABLE 3:** Association of β‐OHB with HFrEF by sex‐stratified tertiles of plasma concentrations of β‐OHB

Events/Participants	T1	T2		T3		β‐OHB Per 1 SD Increment	
♀	5/1042	12/1023		20/1041		37/3106	
♂	21/1015	27/998		52/1015		100/3028	
		HR (95% CI)	*P*‐value	HR (95% CI)	*P*‐value	HR (95% CI)	*P*‐value
Crude Model							
All	(ref)	1.53 [0.93‐2.52]	.09	2.56 [1.63‐4.01]	<.001	1.48 [1.22‐1.79]	<.001
♀	(ref)	2.02 [0.70‐5.84]	.19	3.46 [1.29‐9.29]	.01	1.58 [1.16‐2.17]	.004
♂	(ref)	1.38 [0.78‐2.44]	.272	2.41 [1.45‐4.01]	<.001	1.53 [1.20‐1.96]	<.001
Model 1							
All	(ref)	1.20 [0.73‐1.97]	.48	1.79 [1.13‐2.82]	.01	1.30 [1.06‐1.59]	.01
♀	(ref)	1.75 [0.60‐5.08]	.30	2.97 [1.10‐8.01]	.03	1.54 [1.11‐2.14]	.01
♂	(ref)	1.01 [0.57‐1.80]	.97	1.46 [0.87‐2.45]	.15	1.25 [0.96‐1.62]	.10
Model 2							
All	(ref)	1.09 [0.66;1.80]	.75	1.61 [1.01;2.56]	.05	1.29 [1.04;1.59]	.02
♀	(ref)	1.97 [0.67;5.77]	.21	3.23 [1.17;8.93]	.02	1.53 [1.08;2.17]	.01
♂	(ref)	0.80 [0.45;1.43]	.45	1.17 [0.69;1.99]	.56	1.22 [0.92;1.61]	.16
Model 3							
All	(ref)	1.13 [0.66;1.92]	.66	1.53 [0.93;2.52]	.09	1.26 [1.01;1.58]	.04
♀	(ref)	3.42 [0.91;12.89]	.06	4.75 [1.33;16.91]	.01	1.83 [1.16;2.91]	.01
♂	(ref)	0.80 [0.43;1.47]	.47	1.09 [0.62;1.91]	.76	1.13 [0.88;1.46]	.33
Model 4							
All	(ref)	0.75 [0.42;1.35]	.34	0.95 [0.55;1.63]	.84	1.17 [0.93;1.47]	.17
♀	(ref)	2.09 [0.52;8.45]	.30	2.20 [0.57;8.56]	.25	1.52 [0.93;2.51]	.09
♂	(ref)	0.59 [0.30;1.18]	.13	0.77 [0.41;1.42]	.39	1.10 [0.85;1.42]	.48

Note

Model 1. Age + sex (sex‐specific analyses are adjusted for age only).

Model 2. Age + sex + BMI + T2D + hypertension + myocardial infarction + smoking + alcohol consumption

Model 3. Age + sex+BMI + T2D + hypertension + myocardial infarction + smoking + alcohol consumption + total cholesterol + HDL‐C + triglycerides + leucine + glucose + eGFR + UAE.

Model 4. Age + sex + BMI + T2D + hypertension + myocardial infarction + smoking + alcohol consumption + total cholesterol + HDL‐C + triglycerides + glucose + eGFR + UAE + history of atrial fibrillation + heart rate + NT‐proBNP.

Abbreviations: BMI, body mass index; eGFR, estimated glomerular filtration rate; HDL‐C, high‐density lipoprotein cholesterol; HF, heart failure; NT‐proBNP, N‐terminal pro‐brain natriuretic peptide; T2D, type 2 diabetes; UAE, urinary albumin excretion.

In the same model (Model 3, Table 3), β‐OHB concentrations were associated with increased risk of HFrEF in women, showing a HR of 4.75 (95% CI: 1.33‐16.91; *P* = .01) of the highest tertile vs. the lowest. On the other hand, in men the association was significant in the crude model, but not after the same adjustment (HR: 1.09, CI: 0.62‐1.91, *P* = .76) (Table 3). In a model further adjusted for atrial fibrillation, heart rate and NT‐proBNP, the association remained nonsignificant. Excluding patients with reported T2D at baseline did not meaningfully change the results neither in women (Table S3).

### 3.5 Longitudinal analyses on HFpEF

The analysis of β‐OHB as continuous variable did not reveal a significant association, neither in men (HR per one SD increase: 1.36, 95% CI: 0.95‐1.95; *P* = .09), nor in women (HR per one SD increase: 1.25, 95% CI: 0.91‐1.71; *P* = .17) in the crude model nor in the fully adjusted model. Also in Cox regression analysis that examined the β‐OHB as a categorical variable, plasma concentrations of β‐OHB were not significantly associated with risk of HFpEF in women, neither in the crude model (HR:1.79, CI: 0.80‐3.99, *P* = .15), nor after full adjustment (HR: 0.89, CI:0.18‐4.39, *P* = .88). On the other hand, in men the association was significant only in the crude model (HR: 2.36, CI: 1.04‐5.34, *P* = .03), but not after full adjustment (HR: 1.12, CI: 0.26‐4.77, *P* = .87). (Table S4). Excluding patients with reported T2D at baseline did not meaningfully change the results (Table S5).

### 3.6 NT‐proBNP stratified analysis

The prospective associations of β‐OHB with development of overall HF, HFrEF and HFpEF were evaluated using NT‐proBNP stratified analysis. In the subset of males and females with circulating concentrations of NT‐proBNP below the median value (women < 55 ng/L and men < 32 ng/L), there was no significant association. Contrarily, in individuals with NT‐proBNP levels above or equal to the median value presented a significant association between (HR = 1.19, CI: 1.00;1.42, *P* = .04). This association was more evident in women (HR = 1.52, CI: 1.12;2.07, *P* = .008), and it was not significant in men (*P* = .47) (Table S6).

## 4 DISCUSSION

In the present study, we evaluated whether β‐OHB was prospectively associated with HFrEF or HFpEF in the general population. Older age, female sex, high blood pressure, reduced eGFR, elevated UAE and NT‐proBNP levels were all positive associated of β‐OHB. Remarkably, there was a robust positive association of high concentrations of β‐OHB with incident HF in women, which remained significant after adjustment for established risk factors, including age, BMI, T2D, lipids, cigarette smoking and alcohol consumption as well as eGFR and albuminuria. In contrast, such an independent association between higher circulating β‐OHB with and‐onset HF was not present in men in such adjusted analysis.

To the best of our knowledge, there are no population‐based studies in which plasma concentrations of ketone bodies have been analysed in the context of new‐onset HF. So far, this association has been explored in one study that was comprised of 405 dialysis patients, which were followed for 3.2 years. In this report, high β‐OHB concentrations were associated with risk of CVD events in elderly participants and sex differences were not reported.^12^ In addition, high β‐OHB concentrations were associated with a reduced ejection fraction in subjects with HF,^13^ findings which are in line with the results reported in the present study. Higher concentrations of β‐OHB were found in participants with T2D, as expected.^19^, ^24^ Remarkably, the prospective association of β‐OHB with HF in women was independent of prevalent T2D.

To date, the mechanisms behind the relationship between elevated ketone bodies and HF have not been fully elucidated. It has been shown that cardiac muscle of HF individuals is subject to a gene regulatory shift, resulting in overexpression of 3‐hydroxybutyrate dehydrogenase 1 and , succinyl‐CoA 3‐oxoacid CoA transferase; enzymes which enhance the ketone oxidation pathway, as well as monocarboxylate transporter 1(Slc16a1), which increases the ketone transport capacity.^4^, ^11^ The uptake of ketone bodies in the heart depends on proton‐linked monocarboxylate transporters (MCT), particularly the proteins, MCT1, MCT2, MCT4.^25^ It has been reported that MCT1 is overexpressed in cardiac tissue of murine models with congestive heart failure.^26^ Remarkably, a recent in vivo human study had reported that the uptake of ketones in the heart of HFpEF and HFrEF at least in part depends on circulating concentrations of ketones.^27^ Under the light of these pieces of evidence, and, in the absence of proof of causal relationships, the elevated concentrations of β‐OHB in patients at higher risk of development of HF could be interpreted as the result of a fuel shift, already operative in an early failing heart. In line with this supposition, our findings suggest that β‐OHB was associated with incident HFrEF in subjects with higher circulating NT‐proBNP at baseline.

In addition, the use of recently developed bioenergetics assay platforms allowed the study of mitochondrial respiratory flux on freshly isolated heart mitochondria. This method has led to the conclusion that the heart utilizes β‐OHB as a metabolic stress defence.^28^ Such observations have reinforced the concept that a high plasma concentration of β‐OHB could have a beneficial effect on cardiac performance.^4^ From a pharmacological point of view, it is also noteworthy that one of the proposed mechanisms of SGLT2 inhibition treatment to ameliorate the risk of HF may be related to its ability to increase ketone body availability to the myocardium.^29^

Plasma concentrations of β‐OHB are determined by the interplay of hepatic ketogenesis and ketone body utilization in extrahepatic organs.^3^ Therefore, the baseline elevated concentrations of β‐OHB presented in the participants who developed HF could be attributable to an increased hepatic ketogenesis or a decreased ketone body utilization in extrahepatic tissues. Interestingly, it has been reported that ketone body utilization in skeletal‐muscle is reduced among HF patients compared to healthy control subjects.^30^ Furthermore, it has recently been shown in the mouse model Myh6‐Cre: Dsp^w/f^ that ketogenesis occurs in heart muscle before the development of HF, during early stages of arrhythmogenic cardiomyopathy.^31^

The current study revealed a difference in the association of β‐OHB with HF between men and women. Besides the well‐known differences in the clinical presentation of HF between men and women, with preserved ejection fraction being more frequently observed in women,^32^ ketone body metabolism also shows important sex‐based differences with a higher production of β‐OHB in women.^33^ It has been reported that women have higher fasting plasma ketone body concentrations than men, as well as in response to an oral fat‐loading test.^33^ There are also notorious sex differences in the metabolism of ketogenic substrates, that is leucine. For instance, it has been reported that the net leucine balance in fasting and fed conditions are higher in women compared with men.^34^ Whether the association between branched chain amino acids, including leucine and development of HF,^35^ and other cardiovascular events is mediated by ketone body metabolism remains unexplored.

In addition, it has been proposed that mitochondrial function varies between sexes, with possible consequences for HF development and sex‐specific clinical manifestations.^36^ Recently, it has been shown that women have higher intramyocardial fat concentrations than men. Moreover, it was reported that intramyocardial fat was correlated with left ventricular diastolic parameters (echocardiographic mitral peak velocity of early filling/ early diastolic mitral annular velocity, left atrial volume index and peak filling rate) in women, but not in men with HF.^37^

All these factors could play a role in the sex differences observed related to the association between circulating β‐OHB and HF development. Clearly, the potential role of β‐OHB‐related pathways in the pathophysiology of HF in women merits further investigation.

### 4.1 Strengths and limitations

The present study has several strengths. To the best of our knowledge, this is the first study to report the association between higher plasma β‐OHB concentrations and incident HF in the general population. Furthermore, the large population enrolled in the study enabled us to carry out sufficiently powered multivariable‐adjusted analyses and test the robustness of the findings using several sensitivity analyses to provide solid evidence. Likewise, the long follow‐up time of PREVEND allowed us to study of biomarkers with potentially subtle and cumulative effects, to manifest and impact upon end‐points.

Several limitations of the present study need to be addressed. First, the PREVEND study mainly comprises Caucasian individuals, which could limit extrapolation of these findings to other ethnicities. Second, there are no data on changes in plasma β‐OHB concentrations, and therefore, it is not possible to draw conclusions about the changes on ketone bodies before and after the development of HF. Furthermore, it is worth mentioning that residual confounding is an important limitation of all observational studies. As we performed a prospective epidemiological study, we did not document measurement of β‐OHB concentrations measurements from the aortic root and the coronary sinus to be able to calculate the index of cardiac uptake of ketone bodies or its substrate in our cohort. Due the importance of such data to draw causal conclusions about the role of β‐OHB in the development of HF, further investigation on that regard is required.

## 5 CONCLUSIONS

In this population‐based cohort, it is presented the first evidence that high concentrations of β‐OHB are associated with an increased risk of HF in women. The prospective association was independent of comorbidities. Further investigation is needed to unravel the complexity of the interplay between ketone body metabolism and compromised myocardial function.

## 6 PATIENT AND PUBLIC INVOLVEMENT

No patients were directly involved in setting the research question, developing plans for recruitment, design or implementation of this study. No patients were asked to advise on interpretation or writing of the results. There are no specific plans to disseminate the results of the research to study participants, but the UMCG disseminates key findings from the PREVEND study on its website: https://www.rug.nl/research/portal/datasets/prevention‐of‐renal‐and‐vascular‐endstage‐disease‐prevend(59fb20f0‐8121‐47e8‐acd4‐e9c19041b950).html.

## CONFLICT OF INTEREST

JLFG, BDW, DG, GN, SJLB and RPFD state that they have no conflicts of interest to disclose regarding publication of this article. MAC, IS, EG and JDO are employees of LabCorp. The UMCG, which employs Dr De Boer, has received research grants and/or fees from AstraZeneca, Abbott, Bristol‐Myers Squibb, Novartis, Novo Nordisk and Roche. Dr de Boer is a minority shareholder of scPharmaceuticals, Inc Dr de Boer received personal fees from Abbott, AstraZeneca, MandalMed Inc and Novartis.

## AUTHORS' CONTRIBUTIONS

JLFG, SJLB and RPFD developed and formulated the research questions, have full access to all data in the study and take responsibility for its integrity and the data analysis, wrote the manuscript, contributed to discussion, reviewed and edited the manuscript. BDW, MAC, JDO, DG, IS, EG, GN and RADB contributed to the acquisition of data, contributed to discussion, draft revision and edition the manuscript. All authors approved the final version.

## Funding information

This work was supported by The Dutch Kidney Foundation which supported the infrastructure of the PREVEND program from 1997 to 2003 (Grant E.033). The University Medical Center Groningen supported the infrastructure from 2003 to 2006. Dade Behring, Ausam, Roche, and Abbott financed laboratory equipment and reagents by which various laboratory determinations could be performed. β‐OHB were measured at LabCorp, Morrisville USA at no cost. Dr Flores‐Guerrero acknowledges support from the National Council of Science and Technology (CONACYT).

## Supporting information

Supplementary Material
